# Draft Genome Sequence of *Holospora undulata* Strain HU1, a Micronucleus-Specific Symbiont of the Ciliate *Paramecium caudatum*

**DOI:** 10.1128/genomeA.00664-13

**Published:** 2013-08-22

**Authors:** Hideo Dohra, Haruo Suzuki, Tomohiro Suzuki, Kenya Tanaka, Masahiro Fujishima

**Affiliations:** Instrumental Research Support Office, Research Institute of Green Science and Technology, Shizuoka University, Suruga-ku, Shizuoka, Japana; Department of Biological Science, Graduate School of Science, Shizuoka University, Suruga-ku, Shizuoka, Japanb; Department of Environmental Science and Engineering, Graduate School of Science and Engineering, Yamaguchi University, Yoshida, Yamaguchi, Japanc; National BioResource Project of the Ministry of Education, Culture, Sports, Science and Technology, Chiyoda-ku, Tokyo, Japand

## Abstract

*Holospora undulata* is a micronucleus-specific symbiont of the ciliate *Paramecium caudatum*. We report here the draft genome sequence of *H. undulata* strain HU1. This genome information will contribute to the study of symbiosis between *H. undulata* and the host *P. caudatum*.

## GENOME ANNOUNCEMENT

Bacteria of the genus *Holospora* are intranuclear symbionts of *Paramecium* spp. and are Gram-negative bacteria belonging to the order *Rickettsiales* of the *Alphaproteobacteria* (1–5). *Holospora* currently consists of nine named species, and all species show host specificity and nucleus specificity in their habitats (6). *Holospora undulata* is a micronucleus-specific symbiont of the ciliate *Paramecium caudatum* (7). Here, we present the draft genome sequence of *H. undulata* strain HU1, which provides insight into the symbiotic strategy of this organism.

The infectious form of *H. undulata* cells was isolated from homogenates of the host cells at the early stationary phase of growth, using Percoll density gradient centrifugation (8), and kept until use at -80°C. The draft genome sequence of *H. undulata* HU1 was generated at the Instrumental Research Support Office, Research Institute of Green Science and Technology, Shizuoka University, Japan, using Illumina GAIIx technology. The genomic DNA of *H. undulata* was isolated using a DNeasy blood and tissue kit (Qiagen), fragmented using a Covaris Acoustic solubilizer, and an Illumina GAIIx paired-end (101-bp) library generated 77,451,934 reads totaling 7,822 Mb. The raw sequences were filtered by the FASTX-Toolkit (http://hannonlab.cshl.edu/fastx_toolkit/index.html) to get high-quality reads (cutoff quality score, 20; cutoff read length, <80 bp), resulting in 60,663,426 reads totaling 6,076 Mb. The high-quality reads were then assembled using ABySS version 1.3.5 (9), with a k-mer size of 72 bp, and contigs <200 bp in length were eliminated. The draft genome of *H. undulata* strain HU1 contains 452 contigs consisting of 1,512,931 bp, with a G+C content of 36.2% and an average 4,016× coverage of the total length of contigs.

The draft genome sequence was annotated using the National Center for Biotechnology Information (NCBI) Prokaryotic Genomes Automatic Annotation Pipeline. The annotated genome contains 1,420 protein-coding sequences, of which 650 (46%) are hypothetical proteins without any known functions. Among the 1,420 proteins, 857 proteins (60%) were assigned to different functional categories of NCBI Clusters of Orthologous Groups (COG) (10). Eighty-six proteins were assigned to multiple COG categories. The most abundant COG category was “Replication, recombination, and repair” (186 proteins) followed by “Translation, ribosomal structure, and biogenesis” (125 proteins), and “Cell wall/membrane/envelope biogenesis” (86 proteins). This genome represents a valuable resource for future genomic studies.

### Nucleotide sequence accession numbers. 

The sequencing results are archived in the DDBJ Sequence Read Archive (DRA) database with accession no. DRA001008. The *H. undulata* strain HU1 draft genome sequence has been deposited in GenBank with the accession no. ARPM00000000. The version described in this paper is the first version, ARPM01000000.
